# Parents' Attitude about Hepatitis B Disease and Practice of Hepatitis B Vaccination among Children in Ho Chi Minh City, Vietnam

**DOI:** 10.1155/2019/9323814

**Published:** 2019-07-02

**Authors:** Huynh Giao, Bui Quang Vinh, Nguyen Huynh Tam Lang, Pham Le An

**Affiliations:** ^1^Faculty of Public Health, University of Medicine and Pharmacy at Ho Chi Minh City, Vietnam; ^2^Department of Pediatrics, University of Medicine and Pharmacy at Ho Chi Minh City, Vietnam; ^3^Center for training of Family Medicine, University of Medicine and Pharmacy at Ho Chi Minh City, Vietnam

## Abstract

**Introduction:**

The Expanded Program on Immunization (EPI) in Vietnam for hepatitis B (HepB) among infants has been implemented since 2003. The rates of the birth dose (babies receiving HepB immunization injection within 24 hours after birth) and the later three-dose series were low in 2013-2014.

**Objective:**

This article evaluated attitudes about the hepatitis B disease and vaccine in relation to the correct practice of vaccination among mothers of 12–24-month-old children in Ho Chi Minh City.

**Material and Methods:**

The parents of 768 children aged 12 to 24 months, in Ho Chi Minh City, were interviewed and reviewed their vaccination cards from February 2016 to July 2017.

**Results:**

A total of 768 children had parents of a mean age of 30.8 years, approximately 34% of the children with a mean age of 16.8 months completed all four doses of the hepatitis B vaccine in a timely manner according to the EPI, and only 45.2% of children received the birth dose on schedule within 24 hours. The mother's fears of HepB risk in the community, living in rural areas, and receiving vaccination information from health workers increased the odds of complete and timely HepB vaccination (all p<0.05).

**Conclusions:**

A high rate of children did not receive a complete and timely HepB vaccination in the EPI. Health information strategies should be designed to target urban people and focus on safety of the vaccine, by health workers, to increase the correct practices of hepatitis B vaccination, including the birth dose, and provide education programs that emphasize the high risk for getting hepatitis B.

## 1. Introduction

Hepatitis B virus (HBV) infection is a major global health problem. The World Health Organization (WHO) in 2017 estimated that 325 million people worldwide are living with chronic HBV [1]. In a study among all Asian regions, East Asia (including Vietnam) had the highest prevalence of HBV infection with little change between 1990 and 2005. Generally, endemicity remains high or close to high in this region [2]. Vietnam has high endemic hepatitis B virus (HBV) infection, with 8.6 million people being identified as hepatitis B-positive. An estimated 8.8% of women and 12.3% of men are chronically infected with hepatitis B and the main mode of transmission of HBV in Vietnam is from mother to child (MTCT) during childbirth or early childhood [3]. A survey of unvaccinated children in Thanh Hoa province in 2003 found that current infection (HBsAg+) rates were 12.5% of infants and 18.4% children [4], but a national survey in 2014 showed that the overall prevalence of HBsAg among 6,949 children was 2.70%, and HBsAg prevalence was significantly higher among children born in 2000–2003 (3.64%) compared to children born in 2007–2008 (1.64%), in which HBsAg prevalence among children with ≥3 doses of hepatitis B vaccine including a birth dose (1.75%) was significantly lower than among children with ≥3 doses of hepatitis B (HepB) vaccine but lacked a birth dose (2.98%) and significantly lower than among unvaccinated children (3.47%) [5]. Based on these results, we found the effect of the HepB vaccine for fully immunized infants, compared with nonimmunized infants. The same result was among Chinese children aged 0-9 years, the incidence rate decreasing from 15.86/100,000 in 2004 to 6.36/100,000 in 2010, showed that the prevalence decreased after performing the EPI [6]. The EPI in Vietnam for hepatitis B among infants has been implemented since 2003 [3]. The hepatitis B vaccination schedule was a monovalent HepB vaccine birth dose which was recommended to be given within 24 hours after birth from 2005 [7], and the three-dose series was given as part of a pentavalent DPT-Hib-hepatitis B vaccine (commonly known as QUINVAXEM) scheduled at ages 2 months, 3 months, and 4 months [8, 9]. Coverage of the three-dose series was over 90% between 2011-2014, except in 2013 due to adverse events following immunization (AEFIs) with QUINVAXEM, where the rate dropped significantly to 59% [10]. The birth dose coverage rapidly attained approximately 60% within 2 years after its introduction in 2003 and increased from 65% in 2006 to 75% in 2012. However, the birth dose coverage declined to 55% in 2013 and 2014, following media reports of alleged AEFIs associated with the HepB birth dose administration [11, 12], and slightly increased to nearly 70% in 2015-2016 [13]. Several AEFIs occurred involving both the hepatitis B monovalent vaccine used for the birth dose and the pentavalent vaccine used for the 3-dose series (Quinvaxem) in 2013. These events can cause widespread fears over vaccine safety and reduce the rate of vaccination for children [14]. Parents' decisions to delay or refuse vaccines have been shown to be associated with perceived risks for the HBV infection and safety of the vaccine [15, 16]. For the vaccine program to be effective, the procedures must be acceptable to the parents and they must have a strong belief in its effectiveness to reduce the spread of the disease. We used the Health Belief Model (HBM) (Rosenstock 1966 và Becker 1974) as a theoretical framework to evaluate influences of attitudes about hepatitis B disease and vaccine on the correct practice of vaccination among mothers of 12–24-month-old children in Ho Chi Minh City. The HBM was used to interpret differences in compliant and noncompliant parents with regard to childhood vaccinations. It has been used throughout public health to help explain why people adopt behaviors that lead to healthy lives. The five elements of the HBM include (1) perceived susceptibility to HBV infection (likelihood of getting the disease), (2) perceived severity of HBV infection (perception of how serious an outcome or consequence is from the disease), (3) perceived benefits (efficacy of preventive action undertaken), (4) barriers of vaccination (time, effort, inconvenience, pain, and side effects), and (5) cues to action (information to decide the vaccination) [17, 18].

## 2. Subjects and Methods

### 2.1. Research Design

The study was conducted using a cross-sectional survey.

### 2.2. Research Subjects

A total of 768 eligible fathers, mothers, or caregivers and their children between 12-24 months attended 16 community health centers (CHCs), between February 2016 and July 2017. These CHCs were selected by a simple random approach from 24 Dists in Ho Chi Minh City, then choosing a convenient sample of 48 fathers, mothers, or caregivers and their children from each of the 16 CHCs.

### 2.3. Inclusion Criteria

Research subjects included fathers, mothers, or caregivers and their children aged 12-24 months and a consensus that was approved by their fathers, mothers, or caregivers.

### 2.4. Exclusion Criteria

Parents who did not directly take care of their children were excluded; parents/caregivers and their children were excluded from this study if they did not have a vaccination card.

### 2.5. Data Collection Procedures

A structural questionnaire included three sections. The first section was baseline characteristics of parents/caregivers such as age, gender, residence location, occupation, education, income, members in the household, having infected friends with HBV, attending a health education session on HBV and information of HepB vaccine, and baseline characteristics of children included age, gender, and status of hepatitis B vaccination. The second section assessed parents/caregivers' attitude about the hepatitis B disease and vaccination; a questionnaire included the fifth components of the HBM, combined with Bigham's questionnaires, which was tested for validity and keywords from our preliminary qualitative study [19, 20]. The third section assessed the practice of hepatitis B vaccination among fathers/mothers/caregivers based on vaccination records of their children. The instrument was pretested and subjected to an assessment of construct validity by five experts on immunization in the EPI and public health fields.

The fathers, mothers, or caregivers were interviewed when they took their children aged 12–24 months to the CHCs. Participants were assured that the data collected would remain anonymous.

### 2.6. Variable Definitions

Questions were designed so that each response choice represented an answer a respondent might give if asked the question [21]. In evaluation of attitude about hepatitis B disease and vaccination, for each sampled father/mother/caregiver, we defined the correct attitude when they answered “strongly agree” or “agree” for their attitude about susceptibility to HBV infection, severity to HBV infection, benefits and barriers, and cues to action. Parents were also asked to respond to questions relating to side effects of the vaccine with answers to slight side effects, moderate side effects and serious side effects having a “Yes” response. With regard to the evaluation of practices of hepatitis B vaccination, for each sampled child, we evaluated the vaccination status based on their vaccination records. The binary dependent variables were “timely vaccination” for the hepatitis B vaccine. This was defined as a child received hepatitis B vaccination doses within the schedule proposed by the WHO [22] and recommended by the EPI in Vietnam [9], which was a birth dose and the three-dose series for children aged 2 months (59–88 days old), 3 months (89–118 days old), and 4 months (119–148 days old). In order to measure these, the “time span” for the given vaccination (date of birth subtracted from the date of immunization) was calculated, and timely vaccination was duely concluded if the “time span” fell within the recommended schedule or zero (otherwise). Complete vaccination was defined as a child received full 4 doses of hepatitis B vaccine. Finally, the correct practice was defined if a child was vaccinated both complete and timely hepatitis B vaccine.

### 2.7. Data Analysis

All our estimates were computed using Stata13 and Epidata 3.0 software. Continuous variables were estimated as mean (standard deviation) and discrete variables as frequency and percentage. Comparisons of estimates between 2 groups were performed using the t-test for continuous variables and Chi-square or Fisher's exact test for discrete variables. Multivariate analysis for the binary variable as the practice of correct hepatitis B vaccination was performed using logistic regression with selected variables with significant levels <0.20 in the binary analysis. Statistical p value was defined as <0.05.

### 2.8. Ethical Approval

All subjects agreed and gave informed consent before taking part in the study. This study was approved by the Ethics Council, University of Medicine and Pharmacy at Ho Chi Minh City, Vietnam (protocol number 125/UMP-BOARD). 

## 3. Results

### 3.1. Baseline Characteristics of Parents and Children

A total of 768 parents recruited in the study were mainly in the age group from 25 to 40 years old (85.0%), public workers (35.1%), in high school level education (55.0%), and living in urban areas (73.7%), with only 11.7% parents attending a health education session on hepatitis B. Their children had a mean age of 16.8 months, 51.7% boys, among 65.9% received full 4 doses of the HBV vaccine; only 45.2% of infants received the birth dose on schedule in 24 hours and 33.9% of them got the full and timely vaccination (Table 1).

### 3.2. The Association between HepB Vaccination Status of Children and Baseline Characteristics of Parents and Children

As depicted in Table 2, baseline characteristics of parents/guardians and children receiving both full and timely vaccination were similar to those not getting the full or timely vaccination.

However, children who were living in the rural areas were receiving full and timely vaccinations at a higher rate than children who were living in the urban areas (56.9% versus 25.5%, p<0.001). Children who were living with parents were vaccinated fully and on schedule at a higher rate than those who were living with grandparents (37.0% versus 29.3%, p<0.05) and those whose parent heard about HBV information were vaccinated fully and on schedule at higher rate compared with those whose parents did not (35.1% versus 19.0%, p<0.05) (Table 2).

### 3.3. The Association between HepB Vaccination Status of Children and Parents' Attitude about Hepatitis B Disease

The questionnaire included fourteen items about attitude, three items about prior contact, and two items to estimate vaccination status. Results of responses were summarized in Table 3. We found a statistically significant relationship between the status of hepatitis B vaccination and parent/caregivers' attitude to susceptibility to HepB disease, vaccination information from health workers, and observing serious side effects caused by vaccines (all p<0.05) (Table 3).

Table 4 summarizes results of the logistic regression model of factors associated with getting full and timely HepB vaccine to infants. When adjusted for all other model variables, there was a 2.14-fold increase in the odds of HBV correct immunization for a 1-unit increase in “rural location” (AOR 2.14, 95% CI: 1.77 – 2.59, p<0.001), and there was a 1.24–fold increase in the odds of correct immunization for a 1-unit increase in “thought that their child is at high risk for HBV” (AOR 1.24, 95% CI: 1.01–1.53, p<0.05). In addition, children's parents/caregivers received vaccination information from health workers; their children had a higher rate of full and timely vaccination (AOR 1.31, 95% CI: 1.10 – 1.58, p<0.05).

## 4. Discussion

### 4.1. Low Rate of Full and Timely HepB Vaccination

The results of the study in Ho Chi Minh City found that 65.9% of the 768 children had received four doses of the HepB vaccine, but only 33.9% of children had completed all four doses of the HepB vaccine on schedule and 45.2% received the birth dose in 24 hours according to the EPI. This also indicated that timely birth dose coverage was low. It was similar to Dao Thi Minh An' study on children under five in Vietnam had “timely immunization completion”, among seven vaccines used in the EPI in 2000, 2006, and 2011; hepatitis B dose 1 had the lowest at 17.5%, 19.3%, and 45.5%, respectively [23]. However, it was lower than those in a national survey reported in 2014 and 2016 in Vietnam, the rate of receiving the birth dose with 55.0% and almost 70%, respectively [13]. This could be explained due to the study being conducted in 2016-2017 and included children aged 12-24 months old. Therefore, it correlated with Hep B vaccine coverage in 2014-2015. Therefore, the period of our study was close to the period of the AEFIs in 2013 and the drop in hepB vaccination was consistent.

### 4.2. Factors Associated with the Full and Timely Hepatitis B Vaccination

Our study showed parents in rural areas practiced correct immunization for their children at a higher rate those parents in urban areas (AOR 2.14, 95% CI: 1.77 – 2.59, p<0.001) (Table 4). A similar finding was obtained in a study by Smith (2009), children whose parents neither delayed nor refused vaccination were 1.5 times more likely to live in rural areas (OR 1.5, 95% CI: 1.0 – 2.4, p<0.05) [24]. In addition, parents who received vaccination information from health workers had a higher rate of correct HBV vaccination than those who did not receive it (AOR 1.31, 95% CI: 1.10 – 1.58, p<0.05). This result was also similar to Bigham's study that HBV immunization was significantly associated (p<0.001) with a recommendation for HB immunization from a healthcare professional [19]. Therefore, parents in rural areas were more likely to follow the advice of health commune staff and consequently; their children had a higher rate of correct vaccination over children with parents in urban areas. Health information strategies should be focusing on the safety of the vaccinations and this needs to be delivered by health commune staff to increase the rate of HepB correct vaccination, including the birth dose. Although media influence played a major role in the drop in HepB vaccination in Vietnam, our study did not find a statistically significant difference between groups with and without receiving information via mass media. Our results found a statistically significant relationship between the attitude of parents/caregivers who thought that their children could get sick if they were not vaccinated and the rate of complete and timely HepB vaccination. Parents of the full and timely HBV immunized children had a more active attitude towards high risk for HBV infection than parents of children who did not receive full and timely immunization (AOR 1.24, 95% CI: 1.01–1.53, p<0.05) (Table 4). Yousafzai et al. (2014) also showed a statistically significant relationship between attitudes of HepB disease threats of medical staff and the practices of HepB vaccination. For example, perceived disease threats after exposure to blood and body fluids was a significant predictor of complete HepB vaccination [25]. Smith's study (2009) had similar results showing that parents who delayed and refused vaccines were significantly less likely to believe that their child might get a disease (71.0% versus 90.0%), and their children also had significantly lower vaccination coverage [24]. Therefore, health workers need to inform everyone that all people were susceptible to HBV and their children were also at high risk of getting the disease and need to be fully vaccinated.

## 5. Study Limitations

This study has limitations that should be considered when unvaccinated children were not included in our sample. Therefore, the findings may not be generalizable to all parents with children from 12-24 months of age living in the region. There is also the possibility of social desirability bias; however, interviewers encouraged parents to express their opinions freely. Future studies could be conducted on unvaccinated children.

## 6. Conclusions

The study showed that a high number of children did not receive a full and timely HepB vaccination in the EPI. Health information strategies should be designed to target urban people and focus on the safety of the vaccine. This message needs to be delivered by health workers to increase the rate of the full and timely hepatitis B vaccination, including the birth dose, and provide education programs that emphasize the high risk of getting hepatitis B in the community, including urban areas.

## Figures and Tables

**Table 1:** Baseline characteristics of parents/caregivers and children (n=768).

*Baseline characteristics of parents*	N(%)
Gender (Female)	621(81.0)
Residence location (n=767)	
Urban	565(73.7)
Rural	202(26.3)
Education	
< Primary school	116(15.1)
Secondary school	229(29.9)
> High school	422(55.0)
Occupation	
Government officer/Staff	142(18.5)
Housewife	265(34.6)
Seller/ Retail	70(11.7)
Worker	270(35.2)
Age (years) (M ± SD)	30.8 ± 5.1
<25	68(8.9)
25- 40	652(85.0)
≥40	47(6.1)
Gross household income (n=727)	
Poor, near-poor	36(4.9)
Moderate	691(95.1)
Number of children in the household	
1	375(48.9)
2	318(41.5)
≥ 3	74(9.6)
Members in the household	
Parents	314(40.9)
Grand-parents	454(59.1)
Having infected friends with HBV	138(17.9)
Attended a health education session on HBV	90(11.7)
Having information about HBV	710(92.5)

*Baseline characteristics of children*	

Gender (Male)	397(51.7)
Age (months) (M ± SD)	16.8 ± 4.2
Status of HepB vaccination	
Birth dose vaccination	347(45.2)
Full dose vaccination	506(65.9)
Full and timely vaccination	260(33.9)

**Table 2:** The association between HepB vaccination of children and baseline characteristics of parents and children (n=768).

	Full and timely HepB vaccination		p –value^*∗*^
	Yes (260)	No (508)	
*Baseline characteristics of parent*			
Gender of parents/ caregivers			
Male	48(18.5)	98(19.3)	0.80
Female	211(81.5)	410(80.7)	
Residence location			
Urban	144(55.6)	421(82.9)	*<0.001*
Rural	115(44.4)	87(17.1)	
Education			
< Primary school	43(16.6)	73(14.4)	0.706
Secondary school	77(29.7)	152(29.9)	
> High school	139(53.7)	283(55.7)	
Occupation			
Government officer/Staff	55(22.1)	87(17.8)	0.534
Housewife	88(35.3)	176(36.1)	
Seller/ Retail	30(12.1)	59(12.1)	
Worker	76(30.5)	166(34.0)	
Age (years)			
<25	24(9.3)	44(8.7)	0.960
25- 40	219(84.5)	433(85.2)	
≥40	16(6.2)	31(6.1)	
Gross household income			
Poor, near-poor households	8(3.2)	28(5.9)	0.111
Moderate	243(96.8)	448(94.1)	
Number of Children in household			
1	119(45.9)	256(50.4)	0.470
2	115(44.4)	203(40.0)	
≥ 3	25(9.7)	49(9.6)	
Having infected friends with HBV (*Yes*)	51(19.6)	87(17.2)	0.402
Attended a health education session on HBV (*Yes*)	34(13.1)	56(11.1)	0.408
Members in the household			
Parents	168(64.9)	286(56.3)	*0.022*
Grand-parents	91(35.1)	222(43.7)	
Having information about HBV (*Yes*)	249(95.8)	461(90.8)	*0.013*
*Baseline characteristics of children*			
Gender			
Male	127(48.9)	270(53.2)	0.259
Female	133(51.1)	238(46.8)	
Age (Mean ± SD)	17.0 ± 4.3	16.6 ± 4.1	0.352*∗∗*

*∗*Chi-square and *∗∗*t-test used to compare with and without getting full and timely HepB vaccination groups, excluding missing data.

**Table 3:** Percentages of respondents answering, “strongly agree” or “agree” to Health Behaviour Conceptual Questions and “yes” to prior contact questions, stratified by HepB Vaccination Status of Children (n=768).

*Concepts *	Full and timely vaccination		p -value^*∗*^
	Yes (n=260)	(n=508)	
*Susceptibility to HepB *			
My child is at high risk for HepB	156 (60.0)	233 (45.9)	*<0.001*
I think my child will get HepB in future	218 (83.8)	384 (75.6)	*0.009*
*Severity of HepB*			
HBV is a serious disease	249 (95.8)	477 (93.9)	0.280
My child could be severity if s/he got HepB	209 (80.4)	429 (84.5)	0.155
I'm afraid to even think about my child getting sick with HBV	227 (87.3)	445 (87.6)	0.908
*Benefits and barriers of vaccination*			
Immunization will prevent HBV	244 (93.9)	484 (95.3)	0.399
By getting HepB vaccine, child will not spread HBV to others	189 (72.7)	379 (74.6)	0.567
Need to be HepB vaccination on schedule	228 (87.7)	463 (91.1)	0.132
The HepB shot can be painful	215 (82.7)	418 (82.3)	0.888
Vaccination can cause AEFIs	315 (62.01)	177 (68.08)	0,097
It is a convenient time to take my child in for vaccines	215 (82.7)	406 (79.9)	0.356
It is a convenient location to take my child in for vaccines	217 (83.5)	420 (82.7)	0.784
*Cues to action*			
Vaccination information from health workers	109 (41.29)	166 (32.68)	*0.011*
Vaccination information from mass media	117 (45)	200 (39.37)	0.134
*Prior Contact (n=667)* (yes) *missing 101*			
Observed slightly side effect	89 (34.2)	187 (36.8)	0.481
Observed serious side effect	25 (9.6)	24 (4.7)	*0.009*
My child had side effect after vaccination	44 (27.3)	151 (29.8)	0.542

*∗* Chi-square tests used to the comparison between with and without getting the full and timely hepatitis B vaccine groups, excluding missing data.

**Table 4:** Results of logistic regression factors associated with the full and timely hepatitis B vaccination.

	OR	AOR	95% CI	p-value
Residence location (rural)	2.23	2.14	1.77 – 2.59	*<0.001*
Vaccination information from health workers	1.29	1.31	1.10 – 1.58	*<0.05*
My child is at high risk for hepatitis B	1.46	1.24	1.01 – 1.53	*<0.05*
I think my child will get HBV in future	1.43	1.24	0.94 – 1.64	0.12
Observed serious side effect	1.56	1.25	0.92 – 1.69	0.12

## Data Availability

The primary data used to support the findings of this study are available from the corresponding author upon request.
